# Contextual, Social and Epidemiological Characteristics of the Ebola Virus Disease Outbreak in Likati Health Zone, Democratic Republic of the Congo, 2017

**DOI:** 10.3389/fpubh.2020.00349

**Published:** 2020-08-04

**Authors:** Kathryn E. L. Grimes, Bonaventure Fuamba Ngoyi, Kristen B. Stolka, Jennifer J. Hemingway-Foday, Leopold Lubula, Mathias Mossoko, Antoine Okitandjate, Pia D. M. MacDonald, Amy Nelson, Sarah Rhea, Benoit Kebela Ilunga

**Affiliations:** ^1^RTI International, Durham, NC, United States; ^2^Directorate of Disease Control, Ministry of Public Health, Kinshasa, Congo; ^3^Department of Epidemiology, Gillings School of Global Public Health, University of North Carolina, Chapel Hill, NC, United States

**Keywords:** The Democratic Republic of the Congo, Ebola Virus Disease, outbreak investigation, contact tracing, surveillance, zoonotic disease

## Abstract

While the clinical, laboratory and epidemiological investigation results of the Ebola outbreak in Likati Health Zone, Democratic Republic of the Congo (DRC) in May 2017 have been previously reported, we provide novel commentary on the contextual, social, and epidemiological characteristics of the epidemic. As first responders with the outbreak Surveillance Team, we explain the procedures that led to a successful epidemiological investigation and ultimately a rapid end to the epidemic. We discuss the role that several factors played in the trajectory of the epidemic, including traditional healers, insufficient knowledge of epidemiological case definitions, a lack of community-based surveillance systems and tools, and remote geography. We also demonstrate how a collaborative Rapid Response Team and implementation of community-based surveillance methods helped counter contextual challenges during the Likati epidemic and aid in identifying and reporting suspected cases and contacts in remote and rural settings. Understanding these factors can hinder or help in the rapid detection, notification, and response to future epidemics in the DRC.

## Introduction

In April 2017 (1, 2) the Likati Health Zone office in the northern province of Bas Uélé in the Democratic Republic of the Congo (DRC) identified a cluster of illnesses and deaths with Ebola-like symptoms. Following investigation by local health authorities on May 5, 2017 (3), the Provincial Health Office alerted the Ministry of Health (MOH) in Kinshasa of a potential Ebola Virus Disease (EVD) outbreak, which was subsequently reported to the World Health Organization (WHO) per International Health Regulation requirements (4). The MOH officially declared the EVD outbreak in the Likati Health Zone on May 11, 2017 (1) after a blood sample collected from one of five suspected cases tested positive by reverse transcription-polymerase chain reaction (RT-PCR) (5) for Ebola virus subtype Zaire at the national reference laboratory in Kinshasa (6).

The DRC is the second largest and fourth most populated country in Africa and has an environment favorable to zoonotic disease outbreaks such as yellow fever, monkeypox, EVD, and other viral hemorrhagic fevers (7, 8). Tropical forests rich in animal diversity and growing in population density, like those in the DRC, have been shown to increase the risk of emerging infectious diseases (9). These ecological factors, regional sociopolitical insecurity and instability, shared borders with nine other countries, and a mobile population, make DRC highly vulnerable to disease outbreaks (9, 10). While the DRC is experienced in outbreak response, having responded to more EVD outbreaks than any other country, the current (2018–2019) EVD outbreak in Ituri and North Kivu provinces—the longest-lasting in DRC's history—has demonstrated that when certain factors converge, outbreaks can still be a challenge to contain (11).

The Bas Uélé province—which houses the Likati Health Zone where the May-June 2017 EVD outbreak occurred—is situated in the northern part of the country on the border with the Central African Republic. Likati is a heavily forested, rural area (population of 74,648) (12) ~140 km away from the provincial capital Buta. Likati is isolated and lacks infrastructure, has limited communication networks, and no paved roads; travel routes (dirt paths and rivers) become impassable during the rainy season April through December. These factors result in reduced access to healthcare and delays in detecting, reporting and responding to potential cases of epidemic-prone diseases (13). The poverty rate is high, and the economy is based on agriculture, fishing, and hunting; many rely on the bushmeat industry as a food staple and source of income, increasing risk of zoonotic diseases exposure and transmission (14). The DRC's vulnerability to emerging and re-emerging infectious diseases along with the challenging environmental, geographic and sociopolitical factors renders timely detection and reporting of epidemic-prone diseases difficult, and underscores the importance of continued investment in a strong epidemiological surveillance system to detect and monitor disease outbreaks.

In the 2017 Likati EVD outbreak, the MOH's National Coordination Committee was responsible for managing outbreak response activities, coordinating with national and international partners to develop the outbreak response plan and assembling a multi-sectoral Rapid Response Team (Table 1), which was deployed to the outbreak epicenter on May 13, 2017. As part of the rapid response, the MOH's Directorate of Disease Control was assigned primary responsibility to coordinate the outbreak surveillance and case investigation activities. The Rapid Response Surveillance Team was comprised of field epidemiologists and surveillance experts who were responsible for conducting active case investigations, identifying and monitoring case contacts, tracking case alerts (i.e., symptomatic individuals or unexplained deaths) at health centers and within the community, managing case and contact data, and producing daily Situation Reports. Using the epidemiological, clinical, and laboratory data collected during surveillance activities, the Surveillance Team conducted an epidemiological investigation to identify the chain of transmission, determine the origin of the outbreak, and understand the dynamics of this EVD outbreak. As members of the Rapid Response Team, we describe the methods of our epidemiological investigation and expand upon previously published results (6) by describing the contextual, social and epidemiological factors that contributed to the Likati outbreak, and the potential implications these findings have on future EVD outbreaks in the DRC.

**Table 1:** Overview of Ebola Rapid Response Teams, Likati Health Zone, DRC, May–June 2017^*^.

**Response pillar**	**Description**	**Lead partner**	**Other key partners**
Surveillance team	Organized and implemented active case investigation, contact tracing, and monitoring activities in health center and community. Conducted epidemiological surveillance in the community to trace chain of transmission	MOH-DLM	WHO, RTI
Medical management team	Established Ebola Treatment Centers (ETCs) at Likati general reference hospital and Nambwa Health Center, provided palliative care to suspected cases, educated caregivers and family members on infection prevention	MSF	MOH, WHO, ALIMA
Water and hygiene team	Distributed protective equipment, provided community sensitization on safe burial, implemented infection control activities, installed WASH kits at health structures, public places, and several households	IFRC	UNICEF, WHO
Laboratory and research team	Conducted confirmatory testing in Kinshasa, established mobile laboratories in Likati and Buta, developed testing algorithm, implemented standardized procedures to collect samples from suspected cases at admission, collected second sample as control for negative results, responsible for animal testing	INRB	JICA, WHO
Psychosocial support team	Provided support to suspected cases at ETCs, survivors after they were released, and family members of deceased cases	MSF	ALIMA
Logistics team	Ensured efficient resource management and coordination of staff and materials arriving and departing from Likati and Buta	MONUSCO and WFP	UNICEF, WHO, DFID, USAID
Communication and social mobilization team	Organized awareness-raising activities in villages, schools, markets, and churches. These activities were carried out by CHWs, who used a variety of strategies based on target population (e.g., door-to-door, films, radio, megaphones)	UNICEF	MSF

* *ALIMA, Alliance for International Medical Action; DFID, Department for International Development; DLM, Directorate of Disease Control and Prevention; ETC, Ebola Treatment Center; IFRC, International Federation of the Red Cross; INRB, Institut National de Recherche Biomédicale; JICA, Japanese International Cooperation Agency; KSPH, Kinshasa School of Public Health; MoH, Ministry of Health; MONUSCO, United Nations Organization Stabilization Mission in the Democratic Republic of the Congo; MSF, Médecins sans Frontières; RTI, RTI International; SitRep, Situational Report; UMIR/FARDC, Unité Médicale d'Intervention Rapide/Forces Armées de la République Démocratique du Congo; UNIKIN, University of Kinshasa; WFP, World Food Program; WHO, World Health Organization*.

## Methods

### Case Investigation and Contact Tracing

We visited remote villages throughout the Likati Health Zone to interview case contacts, health workers, traditional healers, community and family members who transported patients, and local authorities to determine how and when the outbreak began. We reviewed health records, investigated unexplained deaths and illnesses in humans and animals, and investigated evidence of animal-to-human transmission of EVD. A standard case investigation form was used to record demographic characteristics; determine methods of exposure; document illness onset and signs and symptoms; and identify potentially exposed contacts of suspect, confirmed and probable cases.

To improve the early detection of suspected cases, we established a community alert system and trained community health workers to rapidly report and effectively manage community alert cases. Based on the outbreak-specific case definitions (Table 2), all alerts in the community were investigated and those meeting the criteria as a suspected case were transported to a health facility for clinical assessment, confirmatory laboratory testing, and appropriate treatment per Integrated Disease Surveillance and Response guidelines (15, 16). Contact information for suspected cases was obtained, and individuals who came in contact with a suspected case in the previous 21 days were defined as case contacts (Table 2). These contacts were monitored by community health workers for 21 days and contacts that began exhibiting symptoms were classified and treated as a suspected case.

**Table 2:** Definitions of alert, suspected, probable, confirmed, non-cases, and case contacts used in the Likati 2018 outbreak (15).

**Classification**	**Definition**
Alert case	Anyone with a sudden onset of high fever OR: bloody urine/diarrhea OR: sudden death
Suspected case	Anyone, alive or dead, presenting or having had a high fever with a sudden onset, and who has been in contact with a suspected, probable or confirmed case of Ebola AND/OR a dead or sick animalOR: Anyone with a high fever with a sudden onset and at least three of the following symptoms: - Headache - Vomiting - Anorexia/loss of appetite - Diarrhea - Intense tiredness - Abdominal pain - Muscle or joint pain - Difficulty swallowing - Difficulty breathing - Hiccups - Skin rash OR: Anyone with unexplained bleeding OR: Anyone dying suddenly and whose death is unexplained OR: Spontaneous abortion
Probable case	Suspected case evaluated by a clinician OR: Deceased case with epidemiological link with a confirmed case OR: Any suspect case that is unable to be confirmed with laboratory testing, but the surveillance team classifies as probable after a case classification meeting there is evidence of an epidemiological link to a confirmed case
Confirmed case	Any suspected case with a positive lab result for viral RNA or antibodies for Ebola (RT-PCR or ELISA)
Non-case	Any suspect case with a negative laboratory result. Non-cases do not have antibodies, RNA, or detectable antigens
Case contact	Anyone who has had contact with a confirmed case or a sick/deceased animal.Contact with a human case is classified as any person who has been in contact with a confirmed case in one or more of the following ways: - Stayed in the same household as the confirmed case in the month preceding symptom onset - Had direct physical contact with the confirmed case (living or dead) during his/her illness - Shared the same means of transport (e.g., plane, boat, vehicle, bike, motorcycle, canoe) - Touched bodily fluids of confirmed case during his/her illness - Handled confirmed case's clothes or linen - Was breastfed by the confirmed caseContact with dead or sick animal is classified as anyone who has been in contact with an animal found dead or sick in at least one of the following ways: - Touched - Handled - Prepared - Touched the blood of an animal - Ate bushmeat

All information on suspected case contacts was aggregated into a contacts list register. Patient information such as identity, method of notification, history of symptoms and treatment seeking behavior, symptoms, laboratory testing, and final classification was aggregated in the case line listing register in Excel. Both registers were uploaded into the Epi Info^TM^ Viral Hemorrhagic Fever (VHF) application, version 0.9.60 (17).

We documented the chain of transmission by analyzing the case investigation forms, the case line listing register, the contacts list register, and transcripts of interviews with EVD cases, survivors, and relatives of deceased cases, extended family, contacts, and community members.

### Classification of Cases

Standard case definitions from the 2011 Integrated Disease Surveillance and Response (IDSR) Technical Guide (16) were adapted for health workers and outbreak response teams to improve assessment and classification of probable, confirmed, or non-cases (Table 2). IDSR alert case definitions were broadened to include any unexplained death **or** anyone with a high fever **or** anyone with bloody diarrhea; previously an alert case was defined as anyone with a high fever **and** bloody diarrhea. Case definitions were posted on health facility walls, and community and facility-based health workers were trained on these definitions to ensure proper classifications in applying case definitions. Community health workers were trained to use the definition for an alert case and notified either the Surveillance Team or health center closest in proximity if an alert case (alive or dead) was identified. Surveillance Team members traveling in the remote health areas and health center personnel were trained to report based on the definition of a suspected case, and would then notify the Rapid Response Team to either transport the patient to receive appropriate medical care or to collect and safely despose of the human remains. Notifications of both alert and suspected cases prompted Surveillance Team investigation; based on the investigation results, alert and suspected cases received a final classification as a probable, confirmed, or non-case according to the specified definitions (Table 2).

## Results

### Case Investigation and Contact Tracing

As previously reported, the outbreak resulted in eight cases, five of which were laboratory confirmed [two by RT-PCR and three by enzyme-linked immunosorbent assay (ELISA)] (5, 18), and three of which were classified as probable. There were four deaths (three men and one woman). Five of the eight confirmed or probable cases came from the Nambwa Health Area (in the Likati Health Zone), which was identified as the outbreak epicenter.

All contacts completed the 21-day monitoring period by June 2, 2017, with no additional cases identified. The WHO officially declared the EVD outbreak over on July 2, 2017, 42 days after the last confirmed case tested Ebola virus-negative the second time; this period, which is twice the maximum incubation period for Ebola virus, is used to confirm the end of human-to-human transmission (19).

### Chain of Transmission and Outbreak Origin

Data suggest that all confirmed and probable cases originated from a single EVD case with bushmeat exposure, and all subsequent cases resulted from human-to-human transmission (6). The epidemiological investigation suggested that the origin of the outbreak began with the index case's contact with bushmeat on March 15, 2017. The index case's brother-in-law, a hunter, brought back a monkey and a wild boar, partially eaten by other wild animals. The investigation uncovered the death of 84 pigs in three villages of the Nambwa health area between March 9, 2017 and May 22, 2017, however testing of the dead pigs by RT-PCR indicated they were not the origin of the outbreak.

The epidemiological investigation found that the index case became symptomatic (with fever, arthralgia and muscle pain, nausea, vomiting) on March 27, 2017, within the incubation period after exposure to bushmeat on March 15, 2017. The index case was treated at a private health facility for ~6 days. The index case experienced hematemesis on April 2, 2017; believing it to be a sign of poisoning, the index case's family brought them to a traditional healer. Showing no signs of improvement after 2 days, the traditional healer referred the patient to a private health center, and upon arrival their temperature was 103.1 degrees Fahrenheit (39.5 degrees Celsius). Symptoms did not improve, and after 1 day of observation they were advised to transfer to the Likati General Reference Hospital. The index case died en route on April 5, 2017, 9 days after symptom onset. Transportation to the hospital was via motorcycle, with a driver and a person assisting with transport. The driver, who later died, was classified as a probable case. The person assisting with transport was classified a confirmed case (serology), and was initially believed to be the index case until the epidemiological investigation, instead, identified them as a contact.

### Classification of Cases

Standardizing case definitions, establishing the community alert system, and training community health workers helped to detect, report, and effectively manage community alerts. Coordination with the Communication and Social Mobilization Team (Table 1) was crucial to ensure alerts were investigated by the Surveillance Team and classified according to standard case definitions; the Communication and Social Mobilization Team organized community awareness campaigns through local radio, churches, market, schools, and other public places to remind the population to report suspected cases or deaths in the community. This collaboration resulted in identifying suspected cases in eight of 11 health areas, with 98 classified as non-cases following laboratory testing and epidemiological investigation. All suspected cases and 583 contacts were monitored for 21 days without any lost to follow-up.

The epidemiological investigation discovered a limited understanding of EVD among community health workers and healthcare facility staff in Likati. In response to this observed gap, we trained 98 community health workers in seven health areas of Likati Health Zone on the EVD community case definition to identify community alerts, case contact identification, data collection and follow-up procedures, and collection of body temperature.

## Discussion

In DRC, previous experience with EVD outbreaks has contributed to improved national preparedness to swiftly coordinate and manage an outbreak response. Decades of experience has led to successful containment strategies that involve both formal health workers, traditional healers, and village social and religious leaders, and substantial efforts have been made in the DRC for capacity-building in epidemiology, laboratory analysis, and patient care, resulting in readily available local expertise that can quickly respond to outbreaks (20). These preparatory efforts contributed to the Rapid Response Team's ability to continually assess and strategically adapt to the evolving situation during the Likati 2017 EVD outbreak. The Surveillance Team succeeded in identifying how and when the outbreak began and developing a detailed description of the chain of transmission, which resulted in effectively interrupting the transmission chain to contain the Likati outbreak in 51 days. The person originally thought to be the index case was determined to be a contact instead; thus, the epidemiological investigation found that the outbreak started on March 27, 2017, a month earlier than was originally reported. Using the adjusted timeline, the MOH outbreak declaration on May 11, 2017 was 45 days after the index case first developed symptoms and was shortly thereafter seen in a private health facilty. Additionally, the epidemiological investigation confirmed that the index case had contact with monkey and wild boar bushmeat. While monkeys are known animal reservoirs for EVD, a wild boar has not been a documented likely orgin of a previous EVD outbreak in the DRC (21).

Understanding the contextual factors that contribute to notification delays may allow for targeted improvement of the surveillance system in DRC in preparation for future EVD outbreaks. In the 2017 Likati EVD outbreak, first responders identified several factors that contributed to the delays in detection and reporting: the use of traditional healers as first-line healthcare and treatment, insufficient knowledge of EVD case definitions at the health center and among community health workers, lack of community-based surveillance systems and tools, and remote rural geographic characteristics.

### Use of Traditional Healers

Interviews with key informants during the epidemiological investigation found that EVD cases—including the index case—received care from traditional healers, which can result in delayed detection of a potential epidemic and the coordinated response necessary to halt viral transmission (22). Traditional healers are often the first point-of-care in rural areas where access to the formal healthcare system may be limited, or when one believes an illness is spiritual and cannot be cured with a medical intervention (23). To improve healthcare linkages for populations in rural settings, the DRC MOH put a national program of traditional medicine in place in 2001 to help regulate care provision in rural areas; however, for various reasons including mistrust between traditional and modern practitioners, traditional healers were not integrated into the national healthcare system (24). Currently, due to the informal nature in which traditional healers operate, they can be difficult to identify for EVD control measures. Despite this challenge, it is critical that future EVD communication campaigns sensitize traditional healers (and private health facilities, where the index case first received treatment) to recognize symptoms and refer sustected cases. Of note, among the eight confirmed and probable EVD cases in the Likati outbreak, only one was determined to be exposed at a healthcare facility and the remaining seven were most likely exposed to the virus in the community. This is an important finding because exposure to EVD in healthcare facilities can lead to rapid amplification of an outbreak as was demonstrated in the 1995 Kikwit outbreak where 25% of cases were among health workers exposed in a healthcare facility (25). Proper infection prevention procedures by healthcare workers at the affected healthcare facility may have also contributed to more rapid containment of the Likati outbreak.

### Community and Facility Health Worker Knowledge

Despite treatment at two local healthcare facilities and a traditional healer, the index case was not properly diagnosed with EVD, leading to a substantial delay in notification of the case. This points to the importance of health workers and communities' ability to recognize the signs and symptoms of EVD. The Surveillance Team observed a limited understanding of EVD among facility-based and community-based health workers in remote health areas of Likati Health Zone; as care seeking from traditional healers or religious leaders often replaces or precedes the formal healthcare system, community health workers should be routinely trained to detect unusual health events in their communities (including suspected EVD cases) and report these events to health authorities. The Surveillance Team's knowledge of this factor led to targeted training of community health workers during the Likati epidemic, emphasizing the important role of community-surveillance systems in remote and rural settings.

### Community-Based Surveillance Systems and Tools

The Likati epidemic demonstrated the important role of communities in contributing to EVD response efforts. Training and equipping community and facility-based health workers with the tools to collect, manage, and properly report community alerts and suspected cases in line with national and international surveillance rules and regulations is critical to containing epidemics. Tools such as low-literacy flip charts and posters with visual depictions of case definitions and data collection and reporting forms, should be standardized and available for use in both the informal and formal healthcare system to bridge the gap between event- and indicator-based surveillance systems (26). To be most effective, the community-based surveillance system in the DRC should incorporate notifications and reporting of Ebola-like symptoms and suspected deaths from traditional healers in the communities. The Likati community alert system developed by the Surveillance Team aimed to address this gap, especially in the more remote health areas in Likati that were far from formal healthcare structures.

### Remote and Rural Geographic Characteristics

Likati's remote and rural geography presented challenges that impacted the ability to conduct outbreak investigation and response activities. Impassable roads and poor network coverage affected timely and accurate communication and reporting from remote health areas. Limited transportation infrastructure between Likati and the general reference laboratory in Kinshasa slowed the diagnosis of initial suspected cases until a mobile laboratory unit could be deployed. To address these challenges, Rapid Response Teams (Table 1) used canoes and motorbikes to traverse rivers and difficult terrain inaccessible by car, brought generators to address inconsistent power supply in the district health office, and used Very Small Aperture Terminal (VSAT) satellites and satellite telephones with solar chargers for connectivity in remote health areas. Further, the Surveillance Team placed satellite phones in communities deemed high-risk to ensure direct, real-time case reporting. Nevertheless, Likati's challenging geographic characteristics may have contributed to the confinement of the EVD outbreak and reduced the risk of transmission to more densely populated urban areas in neighboring health zones (6, 13). The low population density limits human contacts, and lack of infrastructure decreases chances of EVD rapidly spreading between large cities. This is in stark contrast to the 2014-2015 EVD epidemic in West Africa.

## Conclusion

The context in which an EVD outbreak occurs can contribute delays in detection, notification, and rapid response. The 2017 Likati outbreak response was a success; despite delays in notification, the Rapid Response Team successfully worked together to contain the EVD outbreak. Case investigation and contact tracing efforts provided important information about how and when the outbreak began, confirmed the true index case, and developed a comprehensive chain of transmission. The investigation also highlighted epidemiological characteristics that can hinder rapid response efforts; understanding these factors that contribute to notification delays allows for targeted improvement of the DRC's surveillance system to best prepare for future EVD outbreaks. Ongoing efforts to identify gaps, and the motivation of the MOH and international community to implement sustainable solutions, may support improved response to and prevent morbidity and mortality from infectious disease epidemics in the DRC and the wider global community.

## Data Availability Statement

The data analyzed in this study was obtained from the Directorate of Disease Control, Ministry of Public Health, Kinshasa, and Democratic Republic of the Congo. The following licenses/restrictions apply: all data requested related to the Likati EVD outbreak investigation will be deidentified prior to sharing. Requests to access these datasets should be directed to Dr. Benoit Kebela Ilunga, kebelailunga@gmail.com.

## Author Contributions

BN, LL, MM, and AO were members of the Rapid Response Surveillance Team responsible for developing the epidemiological investigation plan, conducting the investigation, collecting and analyzing epidemiological data, and establishing the chain of transmission. BI provided technical oversight to the Rapid Response Ream and contributed to the design of the outbreak epidemiological surveillance strategy. KG, KS, JH-F, and PM provided technical assistance for surveillance activities during the outbreak and compiled the findings of the epidemiological investigation for the manuscript. KG wrote the first draft of the manuscript and steadily improved based on comments from KS, JH-F, and PM. All authors reviewed and approved the final manuscript.

## Conflict of Interest

The authors declare that the research was conducted in the absence of any commercial or financial relationships that could be construed as a potential conflict of interest.

## Abbreviations

DLM: Direction de la Lutte contre la Maladie/Directorate of Disease Control
ELISA: Enzyme-linked immunosorbent assay
EVD: Ebola Virus Disease
IDSR: Integrated Disease Surveillance and Response
INRB: Institut National de Recherche Biomédicale/National Reference Laboratory
RT-PCR: Reverse transcription polymerase chain reaction
VHF: Viral Hemorrhagic Fever.

## References

[1] Ministere de la Santé Publique Communication speciale de Son Excellence Monsieur le Ministre de la Sante Publique en Rapport avec la situation epidemiologique du pays. Republique Democratique du Congo (2017). Available online at: https://reliefweb.int/sites/reliefweb.int/files/resources/20170511---communication-de-sem-le-ministre-de-la-sante%CC%81-sur-situation-e%CC%81p…_0.pdf (accessed August 2, 2019).

[2] World Health Organization Dr Oly Ilunga Kalenga, Minister of Public Health, Announces an Outbreak of Ebola Virus Disease in Likati district, Bas-Uélé Province (northern DRC) following confirmation by the National Biomedical Research Institute. Geneva: World Health Organization (2017). Available online at: https://www.afro.who.int/news/dr-oly-ilunga-kalenga-minister-public-health-announces-outbreak-ebola-virus-disease-likati

[3] World Health Organization Ebola Virus Disease. Democratic Republic of the Congo. External Situation Report 1. Switzerland: World Health Organization (2017). Available online at: https://apps.who.int/iris/bitstream/handle/10665/255419/EbolaDRC-1552017-eng.pdf?sequence=1 (accessed May 15, 2017).

[4] World Health Organization International Health Regulations (IHR). Switzerland: World Health Organization (2019) Available online at: https://www.who.int/topics/international_health_regulations/en/ (accessed April 10, 2019).

[5] Cherpillod P Schibler M Vieille G Cordey S Mamin A Vetter P. Ebola virus disease diagnosis by real-time RT-PCR: a comparative study of 11 different procedures. J Clin Virol. (2016) 77:9–14. 10.1016/j.jcv.2016.01.017 26874083

[6] Nsio J Kapetshi J Makiala S Raymond F Tshapenda G Boucher N. 2017 outbreak of ebola virus disease in northern Democratic Republic of Congo. J Infect Dis. (2019) 22701–6. 10.1093/infdis/jiz107 30942884

[7] Ministere de la Santé Publique Secrétariat General Plan de Développement Sanitaire de la Zone de Santé de Likati. Kinshasa: Ministere de la Santé Publique Secrétariat General (2015).

[8] Stolka KB Ngoyi BF Grimes KEL Hemingway-Foday JJ Lubula L Nzanzu Magazani A. Assessing the surveillance system for priority zoonotic diseases in the Democratic Republic of the Congo, 2017. Health Secur. (2018) 16:S44–53. 10.1089/hs.2018.0060 30480506

[9] Allen T Murray KA Zambrana-Torrelio C Morse SS Rondinini C Di Marco M. Global hotspots and correlates of emerging zoonotic diseases. Nat Commun. (2017) 8:1124. 10.1038/s41467-017-00923-8 29066781 PMC5654761

[10] Moore M Gelfeld B Okunogbe A Paul C. Identifying Future Disease Hot Spots. Infectious Disease Vulnerability Index. Santa Monica, CA: RAND Corporation (2016). 10.7249/RR1605 PMC5568150 28845357

[11] Ilunga Kalenga O Moeti M Sparrow A Nguyen VK Lucey D Ghebreyesus TA. The ongoing ebola epidemic in the Democratic Republic of Congo, 2018-2019. N Engl J Med. (2019) 381:373–83. 10.1056/NEJMsr1904253 31141654

[12] Plan National de Riposte à l'épidémie de la maladie a virus Ébola dans la zone de sante de Likati en province du bas-Uélé MNCC Bas-Uele Province: National Ebola Response Plan in Likati Health Zone (2017).

[13] World Health Organization Managing Epidemics: Key Facts About Major Deadly Diseases. Version 1. Switzerland: World Health Organization; (2018). Available online at: https://www.who.int/emergencies/diseases/managing-epidemics-interactive.pdf?ua=1 (accessed April 10, 2019).

[14] Wolfe ND Daszak P Kilpatrick AM Burke DS. Bushmeat hunting, deforestation, and prediction of zoonotic disease emergence. Emerg Infect Dis. (2005) 11:6. 10.3201/eid1112.040789 16485465 PMC3367616

[15] Democratic Republic of Congo Ministry of Health, Directorate of Disease Control Internal Situation Report 7. Definition de cas de Maladie a Virus Ebola a Partir du Mois de Mars. Situation de la réponse à l'épidémie de la MVE à Likati. May 21 ed. Kinshasa: Democratic Republic of Congo Ministry of Health, Directorate of Disease Control (2017).

[16] Organisation mondiale de la Santé, Centers for Disease Control and Prevention Guide technique pour la Surveillance Intégrée de la Maladie et la Riposte (SIMR) dans la Région Africaine. Second edition (2011). Available online at: https://www.afro.who.int/sites/default/files/2017-06/IDSR-Technical%20-Guidelines-2010_French%20_final.pdf (accessed April 10, 2019).

[17] EpiData Software What is EpiData Software? Denmark: EpiData Association. RRID: SCR_008485; 2001. Available online at: http://www.epidata.dk (accessed August 6, 2019).

[18] Ksiazek TG West CP Rollin PE Jahrling PB Peters CJ ELISA for the detection of antibodies to Ebola viruses. J Infect Dis. (1999) 179(Suppl 1):S192–8. 10.1086/514313 9988184

[19] World Health Organization Declaration of the end of Ebola Virus Disease outbreak in the Democratic Republic of the Congo [Internet]. Ebola Outbreak Situation Reports. Switzerland: World Health Organization; (2017). Available online at: http://apps.who.int/iris/bitstream/handle/10665/255798/EbolaDRC-02072017.pdf;jsessionid=52B61BDE9691418191747ECA86EE1CD5?sequence=1 (accessed March 14, 2019)

[20] Maganga GD Kapetshi J Berthet N Kebela Ilunga B Kabange F Mbala Kingebeni P. Ebola virus disease in the Democratic Republic of Congo. N Engl J Med. (2014) 371:2083–91. 10.1056/NEJMoa1411099 25317743

[21] Rosello A Mossoko M Flasche S Van Hoek AJ Mbala P Camacho A. Ebola virus disease in the Democratic Republic of the Congo, 1976-2014. Elife. (2015) 4:e09015. 10.7554/eLife.09015 26525597 PMC4629279

[22] Manguvo A Mafuvadze B. The impact of traditional and religious practices on the spread of Ebola in West Africa: time for a strategic shift. Pan Afr Med J. (2015) 22(Suppl 1):9. 10.11694/pamj.supp.2015.22.1.6190 26779300 PMC4709130

[23] Kasereka MC Hawkes MT. “The cat that kills people:” community beliefs about Ebola origins and implications for disease control in Eastern Democratic Republic of the Congo. Pathog Glob Health. (2019). 113:149–157. 10.1080/20477724.2019.1650227 31387518 PMC6758634

[24] Democratic Republic of Congo Ministry of Health Plan National de Developpement Sanitaire 2016-2020: vers la Couverture Sanitaire Universelle [Internet]. (2016). Available online at: http://www.nationalplanningcycles.org/sites/default/files/planning_cycle_repository/democratic_republic_of_congo/pnds_2016-2020_version_finale_29_avril_2016.pdf (accessed April 27, 2018).

[25] Khan AS Tshioko FK Heymann DL Le Guenno B Nabeth P Kerstiens B. The reemergence of Ebola hemorrhagic fever, Democratic Republic of the Congo, 1995. Commission de Lutte contre les Epidemies a Kikwit. J Infect Dis. (1999) 179(Suppl. 1):S76–86. 10.1086/514306 9988168

[26] Centers for Disease Control and Prevention Global Disease Detection Operations Center: Event-Based Surveillance. Atlanta, GA: Centers for Disease Control and Prevention (2019). Available online at: https://www.cdc.gov/globalhealth/healthprotection/gddopscenter/how.html (accessed April 10, 2019).

