# Supplementary figures and images for: Contextual, Social and Epidemiological Characteristics of the Ebola Virus Disease Outbreak in Likati Health Zone, Democratic Republic of the Congo, 2017
Source: Front Public Health. 2020 Aug 4;8:349. doi: 10.3389/fpubh.2020.00349 (PMC7417652; doi:10.3389/fpubh.2020.00349)

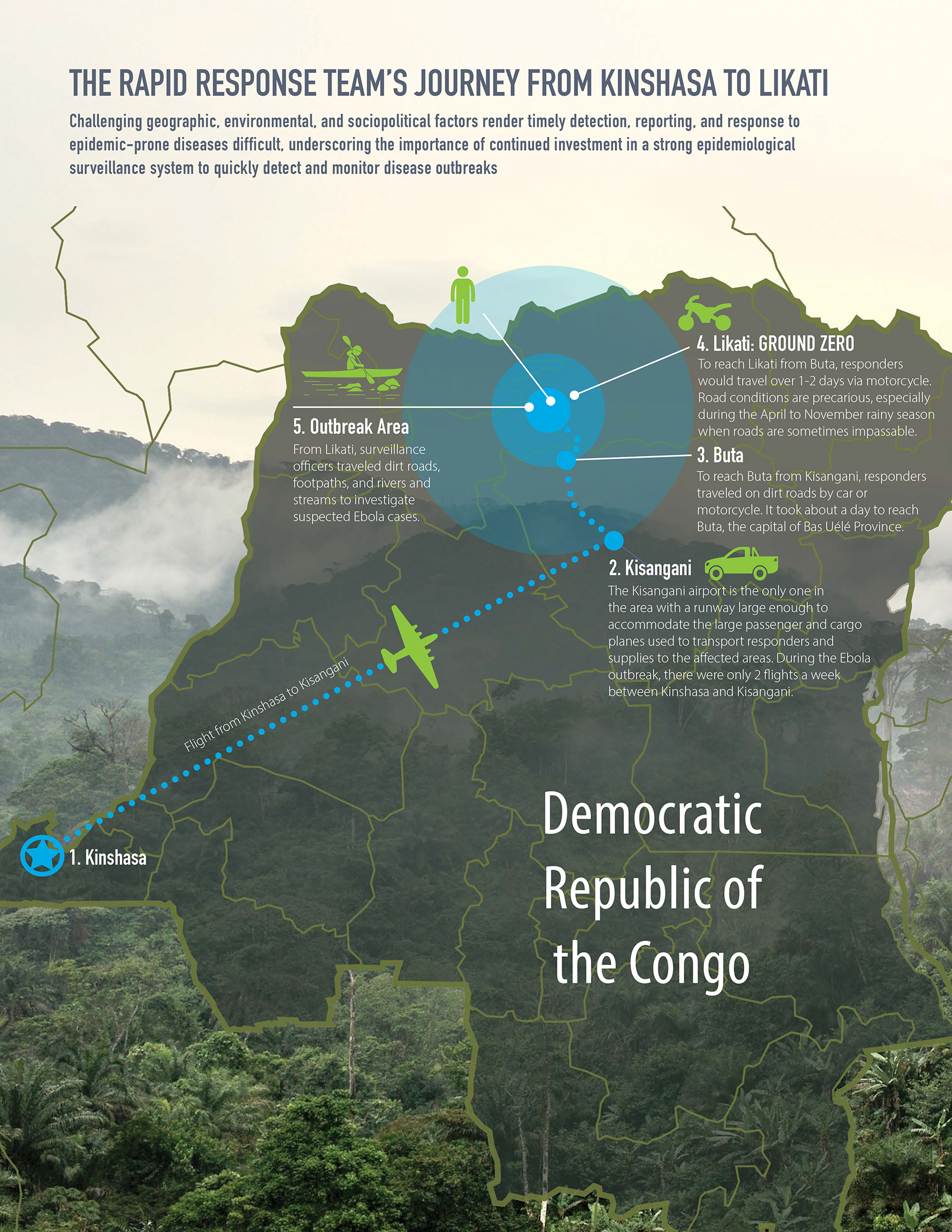

Supplement: Supplementary file 1 [file Image_1.JPEG]
